# Functional status of pediatric patients after extracorporeal membrane oxygenation: A five-year single-center study

**DOI:** 10.3389/fped.2022.917875

**Published:** 2022-08-04

**Authors:** Yang Yuhang, Yang Ni, Zhang Tiening, Wang Lijie, Xu Wei, Liu Chunfeng

**Affiliations:** Department of Pediatrics, Shengjing Hospital of China Medical University, Shenyang, China

**Keywords:** extracorporeal membrane oxygenation (ECMO), new morbidity, Functional Status Scale (FSS), complication, follow-up

## Abstract

**Objective:**

Extracorporeal membrane oxygenation (ECMO) is a widely used treatment for circulatory and pulmonary support in newborns and young children. Over the past decade, the number of children successfully treated with ECMO has gradually increased. However, despite an increasing number of survivors, new morbidity and long-term health issues are becoming more prevalent. A better understanding of the pediatric ECMO prognosis contributes to improved treatment and care programs and minimizes the risk of sequelae and dysfunctions. We aimed to determine the incidence of new morbidity, prognoses, and follow-up data of survivors treated with ECMO in pediatric intensive care units (PICU) using the Functional Status Scale (FSS).

**Methods:**

We retrospectively collected and analyzed clinical data of patients in the PICU who received ECMO from January 2016 to January 2020. Clinical and functional outcomes were assessed at admission and discharge using the FSS. Twenty-seven patients aged between 1 month and 14 years who received ECMO in the PICU were included. Fifty-two percent were male, and the median age was 36 months (interquartile range, 21–114 months). The patients were admitted for fulminant myocarditis (*n* = 13), acute respiratory distress syndrome (ARDS) (*n* = 11), and septic shock (*n* = 3).

**Results:**

This study reviewed a single-center experience using the FSS for ECMO treatment in a PICU. The patients' original conditions included fulminant myocarditis, ARDS, and septic shock. Of the 27 patients who received ECMO, 9 (33%) died, 12 (67%) showed improved condition, and 6 (33%) discontinued treatment and left the hospital. Furthermore, the following adverse events were observed in the survivors who were discharged: nine (50%) cases of lower extremity deep vein thrombosis, seven (39%) jugular vein thrombosis, six (33%) acute kidney injury, five (27%) intracranial hemorrhage and cerebral infarction, and one each of (6% each) pulmonary embolism and peripheral nerve injury. Of the 12 patients who survived through 1 year after discharge, five (42%) recovered completely, whereas seven (58%) showed mild to moderate communication and motor dysfunction. The short-term survival rate and 1-year survival rate of ECMO patients were 67% (18/27) and 44% (12/27), respectively. Additionally, approximately one-third of the patients developed a new morbidity after ECMO treatment (6/18).

**Conclusions:**

High mortality and new morbidity were common in patients who received ECMO treatment. New morbidity increased the risk of death and exacerbated the functional state. Follow-up and rehabilitation after discharge are essential to achieve positive outcomes.

## Introduction

Extracorporeal membrane oxygenation (ECMO) is the most used mechanical circulatory and pulmonary support system in newborns and young children (1). It temporarily takes over the function of the heart and lungs while maintaining the circulation of blood and oxygen to the body. ECMO technology is widely used for cardiopulmonary support, particularly in the field of pediatric and neonatal medicine. Over the past decade, the number of children successfully treated with ECMO has gradually increased. A recent review of the Extracorporeal Life Support Organization (ELSO) data revealed that 30,983 pediatric patients underwent this lifesaving technology from 1990 to 2020 and the ELSO registry reports survival rates for respiratory and cardiac ECMO of 61 and 54%, respectively (2). However, with the rising number of survivors, complications and long-term clinical outcomes have become an important public health concern with reported mortality rates at discharge, 1, 2, and 5 years after ECMO initiation of 40, 45.1, 49.0and 57.4%, respectively, while among the survivors, 21.2% were discharged to continuing care (3). A recent multicenter study found that 56% of the patients who received ECMO treatment survived until hospital discharge, and a new morbidity was found in 40% of the survivors with the Functional Status Scale (FSS), increasing from 6.5 to 9.1 (4). Another study found that the mean FSS of the sepsis patients who received ECMO treatment deteriorated from baseline and the survivors still had problems, such as small acute subarachnoid hemorrhage or massive intraparenchymal hemorrhage centered in the left frontal lobe, and 50% of the survivors needed rehabilitation after discharge (5). Further, lower extremity deep vein thrombosis during ECMO support can lead to the deterioration of motor functional capacity, while intracranial hemorrhage and cerebral infarction may cause cognitive alterations and poor functional outcomes (6, 7). Thus, the ECMO teams should not only focus on the fatality rate and short-term outcomes, but also the long-term sequelae and quality of life after ECMO.

The FSS, an assessment tool for measuring the functional state of patients, is a standardized measure that may reflect the functional status and prognosis. It may be an indicator or a clinical outcome measure in large-scale clinical trials (8, 9). The FSS is based on symptomology scores such as the Pediatric Overall Performance Category Scale, Pediatric Cerebral Performance Category Scale, and the Glasgow Coma Score (10). Owing to the convenience and simplicity of the FSS, it is the most widely used grading system to evaluate the overall prognosis of patients. Currently, insufficient research has been done regarding the use of FSS to assess the morbidity and prognosis of patients who had received EMCO treatment. We report a 5-year single-center study that aimed to determine the incidence of a new morbidity, prognosis, and follow-up data of survivors treated with ECMO in pediatric intensive care units (PICU) using the FSS.

## Materials and methods

### Study population

This retrospective study enrolled all the patients aged between 1 month and 14 years treated with ECMO in the PICU of Shengjing Hospital between January 2016 and January 2020. The inclusion criteria for this study were as follows:

a) Myocarditis

- Cardiogenic shock of various causes: cardiac index less than 2.0 L/min/m2 or left ventricular ejection fraction <30%, systolic blood pressure <90 mmHg, pulmonary capillary wedge pressure ≥ 24 mmHg, dependence on more than two vasoactive drugs at high doses, venous oxygen saturation <55%, and combined with acidosis.
- Cardiac arrest requiring extracorporeal cardiopulmonary resuscitation (ECPR).
- Intractable ventricular cardiac arrhythmia.
- Difficult to remove from extracorporeal circulation.

b) Severe acute respiratory distress syndrome (ARDS): Under optimal mechanical ventilation conditions (FiO2 ≥ 0.8, tidal volume of 6 ml/kg ideal body weight, positive end-expiratory pressure ≥ 5 cmH20, and no contraindications), protective ventilation and prone ventilation are ineffective, and one of the following should be considered as early as possible for veno-venous ECMO support:

- PaO2/FiO2 <5 0 mmHg for more than 3 h.
- PaO2/FiO2 < 80 mmHg for more than 6 h.
- FiO2, 100%; PaO2/FiO2, < 100 mmHg.
- Arterial blood pH < 7.25 and PaCO2 > 60 mmHg for more than 6 h and respiratory rate > 35 breaths/min.
- Arterial blood pH < 7.2 and plateau pressure at respiratory rate > 35 breaths/min. pH < 7.2 and plateau pressure > 30 cmH20 or driving pressure > 15 cmH20.

c) Sepsis, assuming that appropriate volume resuscitation and source control procedures have been completed after the first 6 h of goal-directed resuscitation:

- Inotropic equivalent > 100 with any of the following:
- SvO2 < 55%.
- Lactate > 15mg/dl and not improving.
- Urine output < 0.5 ml/kg/h.

d) ECPR: Those who cannot restore voluntary cardiac rhythm within 15 min of conventional cardiopulmonary resuscitation and who are in cardiac arrest within 1 h.

Exclusion criterion was admission in the PICU lasting <2 h.

### Ethics statement

This study was approved by the Institute Research Medical Ethics Committee of Shengjing Hospital (2021PS654K).

### Definitions

A new morbidity was defined as a worsening of the FSS by ≥3 from the baseline to hospital discharge (6). Functional status at hospital discharge was evaluated among survivors using the FSS, which is composed of six domains (mental status, sensory, communication, motor, feeding, and respiratory) with domain scores ranging from 1 (normal) to 5 (very severe dysfunction). The total scores range from 6 to 30 and are categorized as 6–7 (good), 8–9 (mildly abnormal), 10–15 (moderately abnormal), 16–21 (severely abnormal), and >21 (very severely abnormal) (7).

### ECMO implementation

For venoarterial (VA)-ECMO implementation, venous drainage was initiated from the femoral vein or right atrium with extracorporeal oxygen exchange and then returned to the arterial system *via* the femoral artery (peripheral ECMO) or ascending aorta (central ECMO). For venovenous (VV)-ECMO implementation, venous blood was accessed from the right jugular vein, oxygenated, then returned to the right atrium of the heart after it was made to pass through an oxygenator. The VV-ECMO system comprises a centrifugal pump (ROTAFLOW, Maquet, Germany) and a heat exchanger (Heater-Cooler U35, Maquet, Germany) to maintain a temperature of 37°C. A surgeon, assisted by an operating room team, inserted the ECMO catheters into either an artery or vein. An x-ray was taken to ensure that catheter placement was correct. After the ECMO system was started, heparin (maintenance dose 25–50 IU/kg/h) was used as an anticoagulant. The initial flow rate of ECMO was low but was subsequently regulated to maintain a mean arterial pressure of 65 mmHg. All patients received intravenous cisatracurium (0.1 mg/kg) and intravenous midazolam (2–6 ug/kg^*^min) during ECMO treatment. While on ECMO, the patient was monitored by the PICU doctors and ECMO team.

### Data collection

Trained research coordinators collected all the data *via* a review of the medical records and discussion with bedside clinicians. We retrospectively retrieved the following data: (1) demographic data; (2) incidence of a new morbidity, fatality rate, 1-year mortality, the FSS score at admission, the FSS score at hospital discharge; (3) type of ECMO, duration of ECMO, length of intensive care unit (ICU) stays; and 4) complications that arose during ECMO, categorized as thrombosis, neurologic events, acute renal injury, peripheral nerve injury, and bleeding events. All the enrolled patients were followed up for 1 year after discharge. Eligible patients were contacted by phone.

### Statistical analysis

All statistical analyses were conducted using the IBM SPSS Statistics for Windows, version 20 (IBM Corp., Armonk, NY, USA). Non-normal continuous variables are expressed as median (IQR); categorical variables are expressed as percentages. A *P* < 0.05 was considered statistically significant.

## Results

From January 2016 to January 2020, a total of 5,040 patients were admitted to the PICU of Shengjing Hospital. Twenty-seven patients treated with ECMO were eligible for inclusion in the study population, 52% were male, and the median age was 36 (IQR, 21–114) months. Of the 27 patients, 12 (44%) were discharged with an improved condition, 9 (33%) died, and 6 (23%) abandoned the treatment and left the hospital. The patients' original conditions included fulminant myocarditis (*n* = 13), ARDS (*n* = 11), and septic shock (*n* = 3). Eighteen patients (67%) survived until discharge from the hospital. The incidence of a new morbidity after ECMO treatment was 33% (6/18). The median FSS scores of the 18 survivors at admission and hospital discharge were 15 (IQR, 14–19) and 10 (IQR, 7–30), respectively. Of the 18 survivors, 9 (50%), 7 (39%), 6 (33%), 5 (27%), 1 (6%), and 1 (6%) developed lower extremity deep vein thrombosis, jugular vein thrombosis, acute renal injury, intracranial hemorrhage and cerebral infarction, pulmonary embolism, and peripheral nerve injury, respectively. All the discharged patients reported improvement of function during the follow-up period. However, of the 18 patients who survived to discharge, there were 12 survival and 6 mortality outcomes. Of the 12 patients who survived, only 5 (42%) fully recovered, while 7 (58%) showed mild to moderate motor dysfunction. Additional information is presented in Table 1. The overall survival rate of the 27 initial participants was 67% at discharge and 44% 1 year after discharge.

**Table 1:** Functional Status Scale for original conditions.

**Variables**	**Total Cases**	**ARDS Group**	**Fulminant myocarditis Group**	**Septic shock Group**
	***n* = 27**	***n* = 11**	***n* = 13**	***n* = 3**
**Age, month, median (IQR)**	36 (21–114)	36 (15–54)	108 (36–144)	26 (36–66)
**Sex, Male**, ***n*** **(%)**	14 (52%)	8 (72%)	4 (31%)	2 (66%)
**Weight (kg)**	17 (13–31)	17 (12–18)	33 (16–45)	14 (10–18)
**FSS at admission**				
Mental status	2 (2–3)	2 (2–3)	3 (2–4)	3 (3–3)
Sensory	3 (2–3)	2 (2–3)	3 (2–4)	3 (3–3)
Communication	3 (2–3)	2 (2–3)	3 (2–4)	3 (3–3)
Motor function	1 (1–2)	1 (1–1)	1 (1–4)	1 (1–1)
Feeding	3 (2–3)	3 (3–3)	3(3–5)	5 (4–5)
Respiratory	5 (3–5)	5 (2–5)	5 (3–5)	5 (5–5)
Total FSS	17 (14–20)	15 (13–18)	17 (15–24)	20 (19–20)
**FSS at hospital discharge**				
Mental status	5 (1–5)	5 (1–5)	5 (1–5)	2 (2–4)
Sensory	5 (1–5)	5 (1–5)	5 (1–5)	2 (2–4)
Communication	5 (1–5)	5 (1–5)	5 (1–5)	3 (3–4)
Motor function	5 (2–5)	5 (2–5)	5 (2–5)	2 (2–4)
Feeding	5 (1–5)	5 (1–5)	5 (1–5)	3 (2–4)
Respiratory	5 (1–5)	5 (1–5)	5 (1–5)	1 (1–3)
Total FSS	30 (7–30)	30 (7–30)	30 (7–30)	10 (10–20)
ΔFSS	0 (−9–13)	12 (−9–17)	0 (−8–12)	−8 (−9–1)
**Survival rate**				
At discharge	67% (18/27)	73% (8/11)	62% (8/13)	66% (2/3)
One year after discharge	44% (12/27)	36% (4/11)	46% (6/13)	66% (2/3)
Duration of ECMO (days):	4 (3–5)	4 (3–5)	4 (3–7)	3 (3–4)
Survivors at discharge	18	8	8	2
Survivors at one year after discharge	12	4	6	2
**Lower extremity deep vein thrombosis**				
At discharge	50% (9/18)	50% (4/8)	50% (4/8)	50% (1/2)
One year after discharge	17% (2/12)	25% (1/4)	17% (1/6)	0% (0/2)
**Jugular vein thrombosis**				
At discharge	39% (7/18)	38% (3/8)	50% (4/8)	50% (1/2)
One year after discharge	8% (1/12)	25% (1/4)	0% (0/6)	0% (0/2)
**Intracranial hemorrhage**				
At discharge	22% (4/18)	12.5% (1/8)	12.5% (1/8)	100% (2/2)
One year after discharge	0% (0/12)	0% (0/4)	0% (0/6)	0% (0/2)
**Cerebral infarction**				
At discharge	22% (4/18)	0% (0/8)	25% (2/8)	100% (2/2)
One year after discharge	33% (4/12)	0% (0/4)	25% (2/6)	100% (2/2)

*ARDS, Acute respiratory distress syndrome; ECMO, Extracorporeal membrane oxygenation; FSS, Functional Status Scale; IQR, Interquartile ranges; VA, Venoarterial; VV, Venovenous*.

We compared the clinical data of patients treated with VV-ECMO, VA-ECMO, and ECPR. Patients in the ECPR group had the lowest survival rate. Patients in the VA-ECMO group had the highest rate of hemorrhagic complications (including jugular vein thrombosis, intracranial hemorrhage, and cerebral infarction), which persisted until 1 year after discharge from the hospital (Table 2).

**Table 2:** Functional Status Scale for VV ECMO, VA ECMO and ECPR patients.

**Variables**	**VV ECMO**	**VA ECMO**	**ECPR**
	***n* = 11**	***n* = 12**	***n* = 4**
Age, month, median (IQR)	36 (15–54)	96 (36–126)	68 (4–135)
Sex, Male, *n* (%)	7 (63%)	5 (42%)	2 (50%)
Weight (kg)	17 (12–18)	33 (16–46)	10 (6–19)
FSS at admission			
Mental status	2 (2–3)	3 (2–3)	3 (3–4)
Sensory	2 (2–3)	3 (2–3)	3 (3–4)
Communication	2 (2–3)	3 (2–3)	3 (2–4)
Motor function	1 (1–1)	1 (1–3)	1 (1–2)
Feeding	3 (3–3)	3 (3–5)	3 (3–4)
Respiratory	5 (4–5)	4 (2–5)	5 (5–5)
Total FSS	15 (14–18)	18 (12–21)	18 (17–21)
**FSS at hospital discharge**			
Mental status	3 (1–5)	2 (1–5)	5 (5–5)
Sensory	4 (1–5)	1 (1–5)	5 (5–5)
Communication	4 (1–5)	2 (1–5)	5 (5–5)
Motor function	4 (2–5)	3 (2–5)	5 (5–5)
Feeding	5 (1–5)	3 (1–5)	5 (5–5)
Respiratory	5 (1–5)	1 (1–5)	5 (5–5)
Total FSS	25 (7–30)	11 (7–30)	30 (30–30)
ΔFSS	12 (−9–14)	−1 (−9–10)	13 (9–14)
**Survival rate**			
At discharge	73% (8/11)	75% (9/12)	25% (1/4)
One year after discharge	45% (5/11)	58% (7/12)	0% (0/4)
Duration of ECMO (days):	4 (3–7)	4 (3–5)	3 (3–5)
Survivors at discharge	8	9	1
Survivors at 1 year after discharge	5	7	0
**Lower extremity deep vein thrombosis**			
At discharge	50% (4/8)	56% (5/9)	0% (0/1)
One year after discharge	20% (1/5)	17% (1/7)	0% (0/0)
**Jugular vein thrombosis**			
At discharge	25% (2/8)	56% (5/9)	0% (0/1)
One year after discharge	20% (1/5)	0% (0/7)	0% (0/0)
**Intracranial hemorrhage**			
At discharge	12.5% (1/8)	33% (3/9)	0% (0/1)
One year after discharge	0% (0/5)	0% (0/7)	0% (0/0)
**Cerebral infarction**			
At discharge	0% (0/8)	44% (4/9)	0% (0/1)
One year after discharge	0% (0/5)	57% (4/7)	0% (0/0)

*ARDS, Acute respiratory distress syndrome; ECMO, Extracorporeal membrane oxygenation; FSS, Functional Status Scale; IQR, Interquartile ranges; VA, Venoarterial; VV, Venovenous*.

No significant differences were observed between the survivors and non-survivors in terms of age, sex, weight, primary diagnosis category, mode of ECMO, duration of ECMO, length of hospital stay, and number of complications. The main findings are described in Table 3.

**Table 3:** Univariate analyses of clinical characteristics and survival.

	**Non-survivors**	**Survivors**	***P*-value**
	***N* = 9**	***N* = 18**	
**Age, months, median (IQR)**	96 (36–132)	36 (18–96)	0.253
**Sex**, ***n*** **(%)**			0.785
Male	4 (30.8)	9 (69.2)	
Female	5 (35.7)	9 (64.3)	
**Weight (kg), median (IQR)**	17 (14–28)	17 (12–33)	0.957
**Primary diagnosis category**, ***n*** **(%)**			0.679
ARDS	3 (27.3)	8 (72.7)	
Fulminant myocarditis	5 (38.5)	8 (61.5)	
Septic shock	1 (33.3)	2 (66.7)	
**Mode of ECMO**, ***n*** **(%)**			0.581
VA	6 (37.5)	10 (62.5)	
VV	3 (27.3)	8 (72.7)	
**Duration of ECMO (days), median (IQR)**	3 (3–5)	3 (4–6)	0.706
**FSS at admission**	18 (17–20)	15 (14–18)	0.204
**Length of hospital stay (days), median (IQR)**	16 (5–28)	13 (15 to 24)	0.499
No. of complications^*^, ***n*** **(%)**			0.109
≥ 1	6 (50.0)	6 (50.0)	
None	3 (20.0)	12 (80.0)	

*ARDS, Acute respiratory distress syndrome; ECMO, Extracorporeal membrane oxygenation; FSS, Functional Status Scale; IQR, Interquartile ranges; VA, Venoarterial; VV, Venovenous*.

* *Lower extremity deep vein thrombosis, jugular vein thrombosis, intracranial hemorrhage, and cerebral infarction*.

We compared the groups with a worsening of FSS scores of ≥3 and <3 and found a higher incidence of complications in the group with an FSS score ≥3 than in those with an FSS score <3, among the survivors (Table 4).

**Table 4:** Univariate analyses of clinical characteristics and FSS scores.

	FSS < 3 points^*^	FSS ≥ 3 points^**^	***P*-value**
	***N* = 12**	***N* = 6**	
**Age, months, median (IQR)**	54 (36–120)	15 (12–24)	0.108
**Sex**, ***n*** **(%)**			1.000
Male	6 (66.7)	3 (33.3)	
Female	6 (66.7)	3 (33.3)	
**Weight (kg), median (IQR)**	19 (16–45)	12.5 (10–15)	0.172
**Primary diagnosis category**, ***n*** **(%)**			0.154
ARDS	4 (50.0)	4 (50.0)	
Fulminant myocarditis	6 (75.0)	2 (25.0)	
Septic shock	2 (100.0)	0 (0)	
**Mode of ECMO**, ***n*** **(%)**			0.738
VA	7 (70.0)	3 (30.0)	
VV	5 (62.5)	3 (37.5)	
**Duration of ECMO (days), median (IQR)**	4 (3–5)	6 (3–9)	0.135
**Length of hospital stay (days), median (IQR)**	20 (9–31)	14 (14–15)	0.343
**No. of complications**^**†**^, ***n*** **(%)**			**0.048**
≥ 1	2 (33.3)	4 (66.7)	
None	10 (83.3)	2 (16.7)	

*ARDS, Acute respiratory distress syndrome; ECMO, Extracorporeal membrane oxygenation; FSS, Functional Status Scale; IQR, Interquartile ranges; VA, Venoarterial; VV, Venovenous*.

* *Change in total FSS score < 3 points among survivors*.

** *Change in total FSS score ≥ 3 points among survivors*.

† *Lower extremity deep vein thrombosis, jugular vein thrombosis, intracranial hemorrhage, and cerebral infarction*.

When we compared the clinical data, the functional status upon admission was considerably poorer in the ECMO group than in the non-ECMO group. Second, at the time of discharge, the FSS scores of septic shock patients treated with ECMO were improving, whereas the FSS ratings of the other groups were worsening. Finally, individuals with myocarditis and septic shock who received ECMO had a greater 1-year survival rate than that of patients who did not (Table 5).

**Table 5:** Comparison of FSS scores in ECMO-treated patients and non-ECMO-treated patients.

**Variables**	**ARDS ECMO Group**	**ARDS Group**	**Fulminant myocarditis ECMO Group**	**Fulminant myocarditis Group**	**Septic shock ECMO Group**	**Septic shock Group**
	***n* = 11**	***n* = 14**	***n* = 13**	***n* = 13**	***n* = 3**	***n* =12**
Age, month, median (IQR)	36 (15–54)	29 (8–37)	108 (36–144)	92 (42–144)	26 (36–66)	20 (9–75)
Sex, Male, *n* (%)	8 (72%)	7 (50%)	4 (31%)	5 (38%)	2 (66%)	8 (66%)
**FSS at admission**						
Mental status	2 (2–3)	2 (2–2)	3 (2–4)	1 (1–3)	3 (3–3)	3 (2–3)
Sensory	2 (2–3)	2 (1–3)	3 (2–4)	2 (1–4)	3 (3–3)	3 (2–3)
Communication	2 (2–3)	3 (2–3)	3 (2–4)	2 (1–4)	3 (3–3)	3 (3–4)
Motor function	1 (1–1)	1 (1–1)	1 (1–4)	1 (1–4)	1 (1–1)	1 (1–4)
Feeding	3 (3–3)	3 (3–3)	3(3–5)	5(1–5)	5 (4–5)	3 (3–5)
Respiratory	5 (2–5)	2 (2–2)	5 (3–5)	1 (1–5)	5 (5–5)	4 (2–5)
Total FSS	15 (13–18)	13 (11–17)	17 (15–24)	12 (6–22)	20 (19–20)	16(15–23)
**FSS at hospital discharge**						
Mental status	5 (1–5)	3 (1–3)	5 (1–5)	3 (1–5)	2 (2–4)	5(1–5)
Sensory	5 (1–5)	2 (1–3)	5 (1–5)	4 (1–4)	2 (2–4)	5(1–5)
Communication	5 (1–5)	3 (1–4)	5 (1–5)	5 (1–5)	3 (3–4)	5(1–5)
Motor function	5 (2–5)	1 (1–4)	5 (2–5)	4 (1–5)	2 (2–4)	4 (1–5)
Feeding	5 (1–5)	3 (1–3)	5 (1–5)	5 (1–5)	3 (2–4)	5(1–5)
Respiratory	5 (1–5)	5 (1–5)	5 (1–5)	5 (1–5)	1 (1–3)	5(1–5)
Total FSS	30 (7–30)	17 (6–22)	30 (7–30)	26 (6–29)	10 (10–20)	29 (6–30)
ΔFSS	12 (−9–17)	2 (−5–7)	0 (−8–12)	0 (0–11)	−8 (−9–1)	7 (−8–14)
Survival rate at discharge	73%	86%	62%	69%	67%	83%
Survival rate at 1 year after discharge	36%	36%	46%	38%	67%	42%

*ARDS, Acute respiratory distress syndrome; ECMO, Extracorporeal membrane oxygenation; FSS, Functional Status Scale; IQR, Interquartile ranges*.

## Discussion

In our study, we found that the survival rate at discharge of the patients in the PICU who received ECMO treatment was 67%. Approximately one-third of the patients presented with a new morbidity, which may reflect the severity of illnesses affecting the children receiving ECMO therapy. A recent large multicenter study found that the mortality rate remained high (34.6–50.3%) in those who underwent ECMO (11). Another study reported that 42.4% of the pediatric ECMO patients died prior to discharge, 35.6% had unplanned readmission, and 3% died during readmission within 1 year (12). Studies from the ELSO registry in April 2022 reported survival rates of 61% among pediatric patients who underwent cardiac ECMO, 54% among pediatric patients who underwent respiratory ECMO, and 42% among patients who underwent ECPR (2). The overall survival at our center at discharge is similar to that reported in the ELSO registry; after 1 year of follow-up, the survival rates decreased from 67 to 44%.

Poor physiological status at admission, ECPR, and a new morbidity may account for the high mortality among the patients with ECMO treatment. Compared with the three non-ECMO groups, we found that the ECMO group had higher FSS scores at admission, indicating that the patients in the ECMO group had very low functional status at admission. In addition, survival after ECPR in pediatric patients is poor, and only 25% of patients with ECPR in our center survived till discharge. A previous study found that 54% of the patients who were resuscitated using ECPR did not survive till discharge from the PICU. Half of those who did survive presented with a new morbidity (13). Additionally, all the patients who died within 1 year after discharge had developed a new morbidity, indicating that new dysfunctions may affect the long-term survival outcomes. A retrospective observational study found that after weaning the patients from ECMO, 25.7% died in hospital, and a further 6.9% died in the first 6 months following hospital discharge (14). Besides focusing on the short-term clinical outcomes, increasing attention should also focus on the long-term survival in patients who receive ECMO treatment.

Dysfunctional characteristics vary by specific etiologies. In the fulminant myocarditis group, although the survival rate was above average, the FSS score did not improve over time, while the patients were in the hospital. Additionally, cerebral infarction and hemorrhagic complications contributed to the motor dysfunction. Motor dysfunction was present in 50% of the survivors diagnosed with fulminant myocarditis at discharge from the hospital, whereas at 1 year after hospital discharge, 17% of the patients still experienced motor dysfunction. In cardiac patients supported by ECMO, 18% had significant physical limitations after discharge from the hospital (15).Thus, rehabilitation should remain focused on motor dysfunction in patients with fulminant myocarditis.

In the ARDS group, we found that about 50% of the patients required periodic therapeutic rehabilitation. One study achieved similar results, wherein only one-third of the patients survived, and a substantial proportion of patients required additional hospitalization or hospital services despite surviving to discharge (16). The change in the FSS scores from admission to discharge (ΔFSS) in patients with ARDS was 12 (-9, 17), indicating the presence of functional deterioration during the ICU stay. A previous study found that the median baseline FSS score in patients with pediatric ARDS (PARDS) treated with ECMO increased to 11 (8, 12) at PICU discharge (17). Cashen et al. reported that 29% of patients with PARDS treated with ECMO had moderate (FSS score of 10–15) and severe abnormal functional status (FSS score of 16–21) at hospital discharge (18). Our results are similar to those of these previous studies. After 1 year, the dysfunction rate in the ARDS group decreased to 25%. Patients in the sepsis group had the worst status on admission, of those in the three groups, and the survival rate was much higher than that in the non-ECMO group, one year after discharge. The FSS score at discharge was significantly lower than that at admission, despite the increased incidence of complications. Hence, it is necessary to consider the characteristics of the dysfunction caused by the different diseases, in patients who receive ECMO treatment.

Some potentially serious complications, such as systemic bleeding tendency caused by abnormal coagulation function, as well as cerebral failure caused by massive intracranial hemorrhage, may lead to treatment failure. In a recent ELSO registry report, bleeding complication was independently associated with a high risk of mortality (19). The most common complications were intracranial hemorrhage, epilepsy, secondary infections, and thrombosis according to to ELSO registry report. Additionally, our results revealed that among the survivors, patients with FSS scores ≥3 had a higher incidence of complications. Furthermore, in our study, bleeding complications occurred in 22% of the patients, while thrombosis occurred in 50%; both correlated exactly with vein catheterization and coagulation function. During the 1-year follow-up, as the thrombus disappeared, the motor function gradually improved. Full recovery following intracranial hemorrhage was obtained 1 year after discharge. However, a proportion of patients with accompanying cerebral infarction did not fully recover. A recent study has suggested that 18.1% of pediatric patients with ECMO experience hemorrhagic complications, including intracranial hemorrhage and thrombotic complications (20). We found that communication and motor dysfunctions persisted in patients after discharge. Poor patient outcomes owing to complications remain the focus of ECMO treatment.

The strengths of this study include that this is the first study to evaluate the functional status of patients treated with ECMO based on the heterogeneity of patients with different primary diseases. Furthermore, the present study not only focused on the case fatality rate but also targeted the functional status at discharge and added follow-up information. Moreover, Pollack et al. and the Collaborative Pediatric Critical Care Research Network developed the FSS and confirmed that the scoring system provided a systematic, standardized, and objective assessment independent of a clinician's experience (10). The strengths of this study also include the application of the FSS to evaluate functional status among survivors in the follow-up.

## Limitations

The study has several limitations. First, this was a retrospective study with a small sample size, and it was conducted using data from only one pediatric center. Hence, there is a risk of missing data and information bias. Second, only the type and duration of treatment were analyzed in this study, but other features of treatment, such as therapy intensity, time-points of ECMO intervention, and treatment combinations were unavailable. Third, patients receiving the ECMO treatment may have had poor physiological status upon admission. In such a case, the FSS score would already be relatively high and would not effectively show further deterioration; hence, the rate of a new morbidity may be underestimated. Additionally, the follow-up duration was shorter than that reported in previous studies (15, 18); furthermore, the rehabilitation training information of patients after discharge was not included. Further research should focus on the functional status of patients in a multicenter and longer follow-up clinical trial on pediatric patients with ECMO. For the patients, not only the mortality at discharge but also the functional change after discharge should be focused on.

## Conclusions

This study reviewed a single-center experience using the FSS for ECMO treatment in a PICU. The patients' original conditions included fulminant myocarditis, ARDS, and septic shock. The short-term and 1-year survival rates of ECMO patients were 67 and 44%, respectively. Approximately one-third of the patients developed a new morbidity after ECMO treatment. New dysfunctions may affect the long-term survival outcomes and complications may lead to worsening of the functional state.

## Data availability statement

The original contributions presented in the study are included in the article/supplementary material, further inquiries can be directed to the corresponding author.

## Ethics statement

The studies involving human participants were reviewed and approved by Ethics Committee of Shengjing Hospital Affiliated to China Medical University. Written informed consent from the participants' legal guardian/next of kin was not required to participate in this study in accordance with the national legislation and the institutional requirements.

## Author contributions

YY contributed to formal analysis and original draft preparation. YN and WL contributed to visualization. YY, YN, and WL contributed to methodology. ZT and XW contributed to software and data curation. LC and YY contributed to conceptualization. LC contributed to validation, review and editing, supervision, project administration, and funding acquisition. All authors have read and agreed to the published version of the manuscript.

## Funding

This research was funded by the National Natural Science Foundation of China (No. 81771621) and the Natural Science Foundation of Liaoning Province (No. 2019JH8/10300023).

## Conflict of interest

The authors declare that the research was conducted in the absence of any commercial or financial relationships that could be construed as a potential conflict of interest.

## Publisher's note

All claims expressed in this article are solely those of the authors and do not necessarily represent those of their affiliated organizations, or those of the publisher, the editors and the reviewers. Any product that may be evaluated in this article, or claim that may be made by its manufacturer, is not guaranteed or endorsed by the publisher.
